# Protein Recognition and Assembly by a Phosphocavitand

**DOI:** 10.1021/jacs.5c08121

**Published:** 2025-07-22

**Authors:** Colin P. Wren, Ronan J. Flood, Niamh M. Mockler, Martin Savko, Maura Malinska, Qiang Shi, Peter B. Crowley

**Affiliations:** † School of Biological and Chemical Sciences, University of Galway, Galway H91 TK33, Ireland; ‡ Synchrotron SOLEIL, L’Orme des Merisiers, Saint-Aubin BP 48, Gif-sur-Yvette Cedex 91192, France; § Faculty of Chemistry, 49605 University of Warsaw, Pasteura 1, Warsaw 02-093, Poland; ∥ Advanced Materials Institute, Qilu University of Technology, Shandong Academy of Sciences, Jinan 250014, China

## Abstract

Controlled protein assembly is an enabling technology, in particular, for biomaterials fabrication. Here, we report protein recognition and assembly by a phosphate-containing macrocycle (**pctx**). We show that the *C*_3_-symmetric phosphocavitand is a versatile receptor for N-terminal residues or arginine but not lysine. Using atomic resolution X-ray diffraction data, we reveal the precise details of N-terminal complexation in the β-propeller protein lectin (RSL). In some cocrystal structures, a tetrahedral cluster of the phosphocavitand occupies one end of the β-propeller fold, providing a node for protein assembly. The macrocycle cluster is compatible with different types of precipitants, a broad pH range, and zinc complexation. We demonstrate system control with an arginine-enriched RSL that alters the overall assembly due to selective arginine complexation by **pctx**. A lysozyme–**pctx** cocrystal structure also demonstrates arginine complexation by the macrocycle. An alternative macrocycle cluster occurs with an engineered RSL bearing an extended N-terminus. In this structure, involving zinc ligation at the N-terminus, the macrocycle forms trimeric clusters and four such clusters form cage-like substructures within the tetrahedral protein framework. Thus, N-terminal complexation in combination with phosphocavitand self-assembly provides new routes to protein crystal engineering.

## Graphical abstract

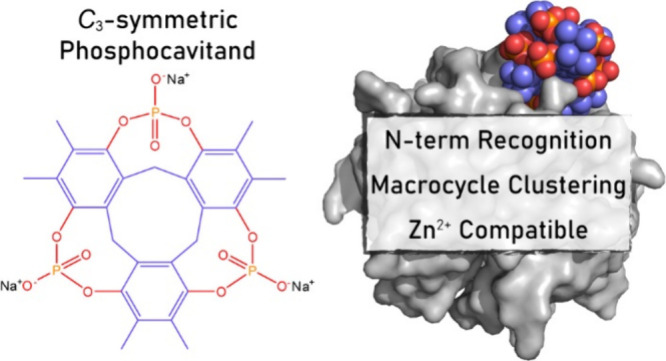

## Introduction

The selective noncovalent modification of proteins by synthetic receptors has diverse applications from sensing to assembly and biomaterials fabrication.^1–5^ Receptors that selectively bind small sites on protein surfaces, including unique features such as the N-terminus, are desirable.^2,4,5^ Here, we describe the protein recognition and assembly properties of a phosphate-containing macrocycle. We show that, in addition to complexing the N-terminus, the phosphocavitand forms clusters that act as nodes for protein assembly. Multiple crystal structures reveal how the macrocycle clusters can engage different protein features, as well as complexing zinc ions, resulting in distinct assemblies. This approach complements the major developments in protein assembly and crystal engineering^2,3^ utilizing computationally designed,^6,7^ metal-mediated,^8,9^ or ligand-mediated interfaces.^4,10–12^

The macrocycle of interest is the recently reported phosphate derivative of *C*_3_-symmetric cyclotrixylohydroquinoylene, dubbed **pctx** and produced in 3 reaction steps.^13,14^ Compared with the bowl-shaped, *C*_4_-symmetric sulfonato-calix­[4]­arene (**sclx**_**4**_, a known protein receptor^4^), the saucer-shaped **pctx** is smaller, shallower, less polar and less anionic (Figure 1, Table 1). While both rims of **sclx**_**4**_ are polar, the lower rim of **pctx** is manifestly hydrophobic. Another distinction between these two hosts is their differing flexibility. The singly bridged **sclx**_**4**_ bowl can breathe and the sulfonate substituents rotate freely. In contrast, the doubly bridged **pctx** saucer is rigidly constrained and each phosphate points one oxygen atom toward the macrocycle center. The resulting cavity has a portal dimension of P–O···O–P ∼ 6 Å. These anionic hydrogen bond acceptors, oriented toward the macrocycle center and delimiting the cavity, appear to be pivotal in guest binding by the shallow **pctx**. Considering the phosphate charge distribution, the −P–O groups are equivalent. For example, the related molecule diphenyl phosphate forms hydrogen bonded ionic chains with imidazole (CCDC 1226648), in which each −P–O group contributes equally.^15^ We note also the intriguing structural similarity between **pctx** and compound 1 in ref 16. This bisaryl phosphate has strong hydrogen bonding capability, again involving equivalent −P–O groups.^16^

**Figure 1:**
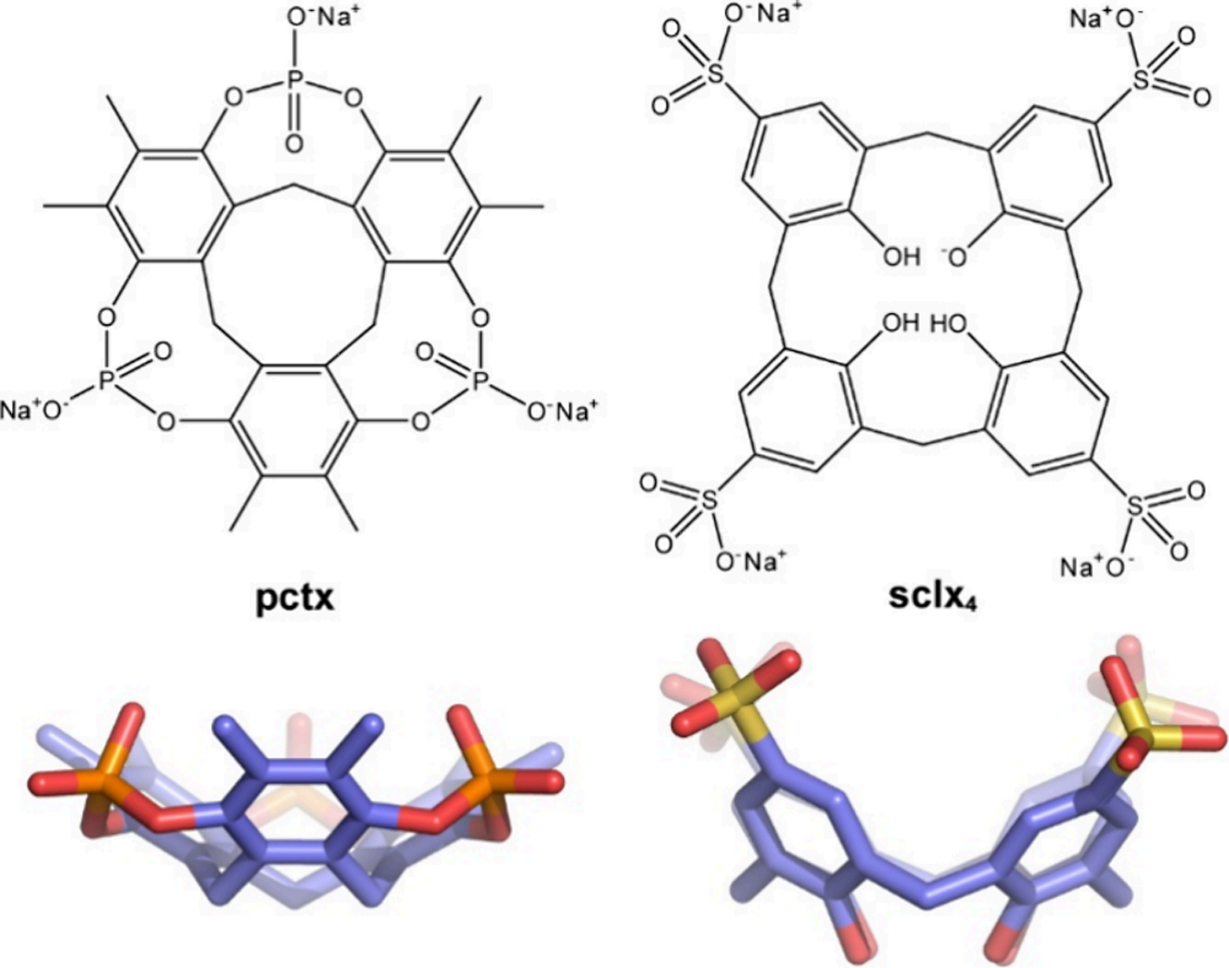
Molecular structures of the **pctx** and **sclx**_**4**_ sodium salts and 3D representations of the corresponding saucer and bowl. Refer to main text for consideration of charge distribution in **pctx**. Color code: carbon in lilac, oxygen in red, phosphorus in orange, sulfur in yellow.

**Table 1:** Physico-Chemical Properties of **pctx** and **sclx**_**4**_

receptor	MW_*t*_ (Da)	surface area (Å^2^)	shape	cavity Ø (Å)	cavity depth (Å)	charge^a^
**pctx**	636	∼670	saucer	∼6	∼3.5	–3
**sclx**_**4**_	745	∼800	bowl	∼6	∼6	–5

a Assuming complete deprotonation.

Considering the utility of **sclx**_**4**_ for N-terminal, lysine or arginine complexation and protein assembly,^4,17–19^ we envisaged that **pctx** would be a useful receptor, especially suited to arginine complexation.^20^

The test protein we studied is lectin (RSL, ∼30 kDa), a 6-bladed β-propeller with *C*_3_ and pseudo *C*_6_ symmetry (Figure 2).^10^ The cocrystallization of **pctx** with RSL and two variants of RSL resulted in five cocrystal structures. Remarkably, in each structure **pctx** binds the accessible N-terminus and forms clusters. The macrocycle clusters are compatible with zinc, an important bridging ion for directing protein assembly.^2,8,9,21^ One of the protein–**pctx**–zinc cocrystals incorporates two types of cages, including a substructure similar to metal organic cages.^8,22^ None of the structures involve lysine encapsulation, and simple modeling reveals why this residue is not a suitable guest for **pctx**. An arginine-enriched variant of RSL (RSL-R_6_)^10^ yields a striking modification of the protein assembly due to **pctx**–Arg complexation. A cocrystal structure with chicken egg white lysozyme^18^ provides further evidence of **pctx**–Arg interactions. This work expands our knowledge of biomolecular recognition by phospho-containing synthetic receptors,^20,23–26^ and provides new components for the protein assembly toolkit.^2,3^

**Figure 2:**
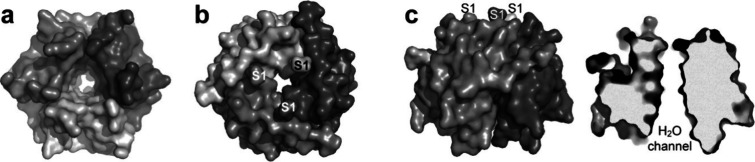
Surface representations of *C*_3_-symmetric RSL showing the toroidal structure and the funnel-shaped water channel, viewed from (a) below, (b) above and (c) the side. A side view cross-section emphasizes the channel. The N-termini (S1) are indicated in panels b and c.

## Results

### Protein–pctx Cocrystallization and Space Groups

A sparse matrix cocrystallization screen of RSL and up to 50 mM **pctx** yielded crystals in six out of 96 conditions (Table 2, Figure 3a–c, and SI Methods). Using these crystals and diffraction data collected at the PROXIMA-2A beamline of SOLEIL synchrotron, we solved three RSL–**pctx** cocrystal structures in space groups *H*3, *P*6_3_, and *H*32 (Tables 2, 3, and S1). All three structures involve the same cluster arrangement of **pctx** (vide infra). The *P*6_3_ and *H*32 structures contain similar RSL–**pctx** assemblies but the latter structure also includes zinc ions. Apparently, the macrocycle cluster is compatible with different types of precipitant (high salt or PEG), a relatively broad pH range, and high concentrations of metal ions such as Li^+^ or Zn^2+^ (Table 2). We obtained two additional crystal forms with variants of RSL. The arginine-enriched RSL-R_6_, with all lysine residues replaced by arginine (Lys25Arg, Lys34Arg, and Lys83Arg),^10^ cocrystallized with **pctx** in space group *H*3 (Figure 3d). The variant MK-RSL, with the macrocycle-binding N-terminal Met-Lys tag,^5,19^ cocrystallized with zinc and a different type of **pctx** cluster in space group *P*4_1_2_1_2 (Figure 3e).

**Table 2:** RSL–**pctx** Cocrytals Obtained in a Commercial Screen and Characterized by X-ray Diffraction^a^

#	precipitant	buffer	additive	space group
C11^b^	2 M ammonium sulfate	0.1 M Na acetate pH 4.6		*H*3
F2	3.15 M ammonium sulfate	0.1 M Na citrate pH 5.0		*H*3
F10	1.1 M sodium malonate	0.1 M HEPES pH 7.0	0.5% Jeffamine ED-2001	*P*6_3_
C2	20% PEG 6000	0.1 M Na citrate pH 4.0	1 M lithium chloride	*P*6_3_
D1	24% PEG 1500		20% glycerol	*P*6_3_
E6^c^	20% PEG 3000	0.1 M imidazole pH 7.8	0.2 M zinc acetate	*H*32

a Alphanumeric code indicates the condition from JCSG HTS++ (Jena Biosciences).

b RSL-R_6_–**pctx** cocrystals (space group *H*3) grew in similar conditions.

c MK-RSL–**pctx** cocrystals (space group *P*4_1_2_1_2) grew in this condition only.

**Figure 3:**
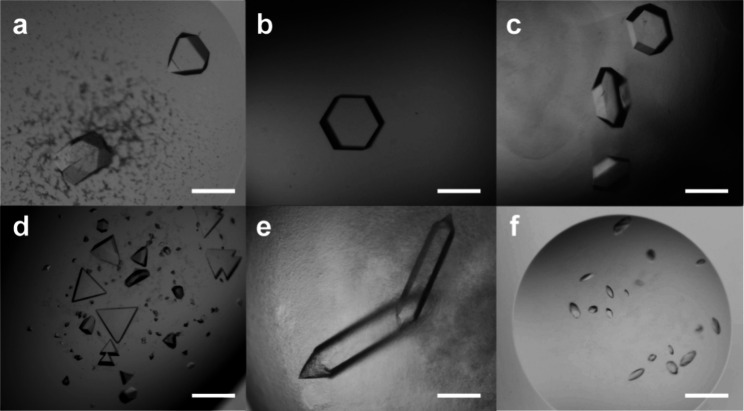
**pctx** cocrystals with (a–c) RSL, in space groups *H*3, *P*6_3_, and *H*32, respectively, (d) RSL-R_6_ in space group *H*3, (e) MK-RSL in *P*4_1_2_1_2 and (f) lysozyme in *P*3_1_21. Two cocrystals (c, e) also contain zinc. Refer to Table 2 for crystallization conditions. Scale bar = 200 μm.

**Table 3:** Unit Cell Parameters of Protein–**pctx** Cocrystal Structures

protein	space group	res. (Å)	*a* (Å)	*b* (Å)	*c* (Å)	%SC^a^	PDB
RSL	*H*3	1.0	46.709	46.709	102.923	40	9HRV
RSL-R_6_	*H*3	1.5	43.569	43.569	122.997	42	9HRZ
RSL	*P*6_3_	1.1	46.078	46.078	139.565	39	9HRW
RSL	*H*32	1.5	45.893	45.893	421.816	39	9HRX
MK-RSL	*P*4_1_2_1_2	1.5	112.158	112.158	99.312	50	9HRY
lysozyme	*P*3_1_21	1.9	86.307	86.307	72.738	53	9HRU

a % solvent content from Matthew’s calculation, accounting for protein and macrocycle masses.

While neutral RSL (isoelectric point p*I* ∼ 6.5) remains soluble in the presence of up to 50 mM **pctx**, the cationic, arginine-rich lysozyme (p*I* ∼ 10) is soluble only up to 5 mM of the anionic macrocycle. Heavy precipitates form at **pctx** concentrations above 5 mM. Sparse matrix crystal screening of **pctx** and lysozyme gave one hit. These unusual ellipsoid crystals, obtained in 1.6 M MgSO_4_, 0.1 M HEPES pH 6.5 (Figure 3f), proved to be a lysozyme–**pctx** complex in space group *P*3_1_21 (Table S2).

### pctx Encapsulates the N-Terminus

RSL–**pctx** cocrystals in space group *H*3 diffracted to atomic resolution and the electron density maps clearly reveal encapsulation of the N-terminus (Figures 4a and S2). ∼115 Å^2^ of Ser1, or ∼65% of the residue, is buried by the macrocycle. Table 4 lists the four noncovalent bonds crystallographically discernible between Ser1 and **pctx**. Ser1-N^α^ forms a salt bridge to one of the phosphates, while Ser1-C^α^ and Ser1-C^β^ each make CH-π bonds with phenyl rings of the macrocycle. The fourth interaction is a hydrogen bond between the Ser1 hydroxyl (O^γ^) and a phosphate of **pctx**. Another phosphate of **pctx** forms hydrogen bonds with the Ser2 amide backbone and the Gln4 side chain. Considering solvation, eight water molecules form hydrogen bonds to the three phosphates (H_2_O···O–P-**pctx** = 2.6–3.1 Å). A ninth water is hydrogen bonded to the N-terminus (Ser1-N^α^···H_2_O = 2.8 Å). Thus, multiple noncovalent bonds mediate the RSL–**pctx** complex. Similar features of N-terminal complexation occur in the *P*6_3_ cocrystal structure, which also includes the N-terminal ammonium making a cation−π bond with **pctx** (Ser1-N^α^···centroid = 3.2 Å). A different type of N-terminal encapsulation occurs with MK-RSL (vide infra).

**Figure 4:**
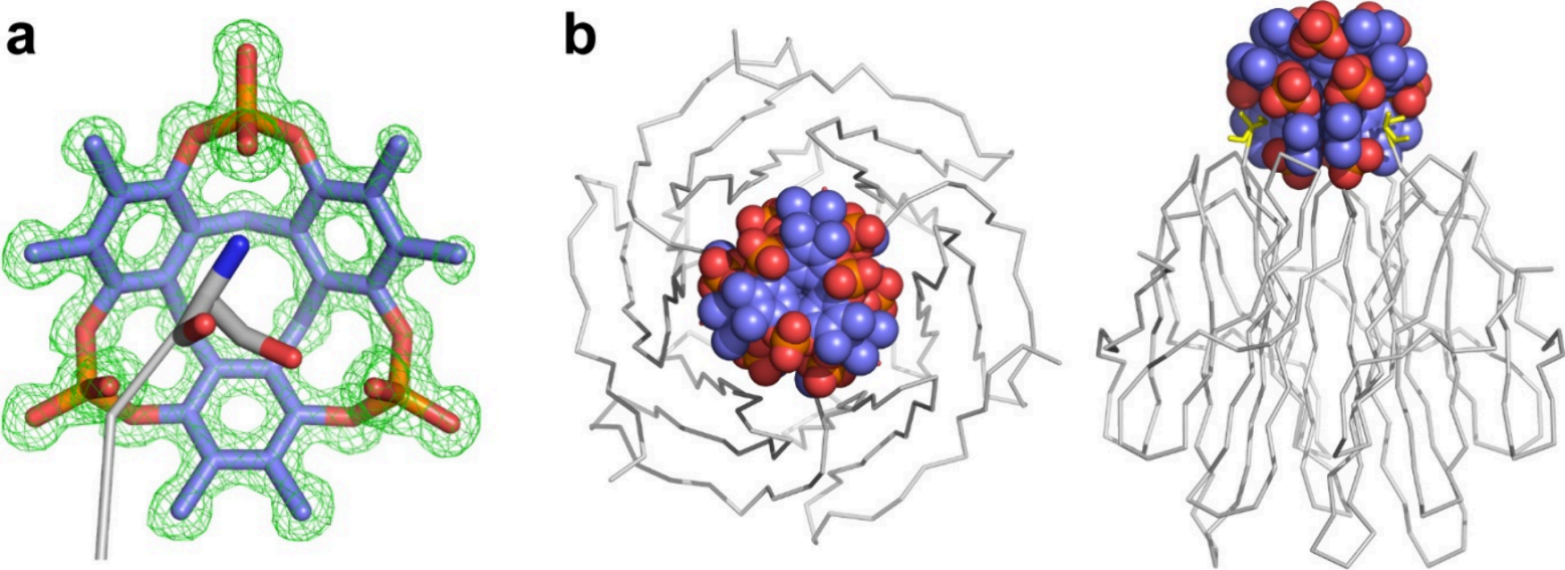
(a) An X-ray crystal structure at 1 Å resolution shows **pctx** encapsulating Ser1 of RSL. The refined 2Fo–Fc electron density map (green mesh) is contoured at 1σ. (b) **pctx** forms a tetrahedral cluster located at the narrow end of the RSL β-propeller trimer. Shown are two views of the assembly, related by 90° rotation, with **pctx** as spheres and RSL as C^α^ trace. Ser1 highlighted in yellow. Note the 3-fold phosphate junctions in the **pctx** cluster.

**Table 4:** RSL–**pctx** Noncovalent Bond Lengths Determined in a 1 Å Resolution Crystal Structure

residue-atom	pctx^a^	distance (Å)	interaction
Ser1-N^α^	O–P^1^	2.7	salt bridge
Ser1-C^α^	centroid	3.8	CH−π (cation−π)
Ser1-C^β^	centroid	3.4	CH−π
Ser1-O^γ^	O–P^2^	2.5	hydrogen bond
Ser2-N^α^	O–P^3^	2.8	hydrogen bond
Gln4-N^ε^	O–P^3^	2.9	hydrogen bond
Lys34-N^ζ^ ^b^	O–P^2^	2.8	salt bridge
Tyr37-centr^b^	Me	3.7	CH−π

a O–P^X^ indicates arbitrary numbering of the three phosphates.

b Residue from another RSL (symmetry mate) in the crystal packing.

### Plugging the Toroid with a Macrocycle Cluster and Assembly Node

β-propeller proteins are toroidal like a Bundt cake.^10,27^ In trimeric RSL, the central water-filled channel is funnel-shaped, with a wide end and a narrow end (Figure 2). The three solvent exposed N-termini are collocated at the narrow end. Each N-terminus of RSL binds a macrocycle, the three of which form the base of a tetrahedron (Figure 4b). A fourth capping macrocycle completes the **pctx** tetrahedral cluster and is devoid of a guest. The tetrameric **pctx** cluster plugs the narrow end of the RSL β-propeller toroid (Figure 4b). In addition to the RSL binding at the base of the **pctx** tetrahedron, three other RSL trimers assemble on the periphery of the **pctx** cluster.

The 3-fold rotational axes of the tetrahedral cluster comprise three phosphates, one from each **pctx**, flanked by pairs of methyl substituents (Figure 4b). Cationic features on the protein complement these anionic patches of the cluster, contributing to charge neutralization in the crystalline assembly. Depending on the crystal form, different cationic features of RSL engage **pctx** in *exo* interactions (Figure 5). With a 6-bladed β-propeller topology and pseudo *C*_6_ symmetry (Figure 2), RSL can pack in layers with a honeycomb-like arrangement. The *H*3 cocrystal comprises protein layers interspersed by **pctx** clusters nestling in the approximately tetrahedral interstitial sites (Figure 5a). The Lys34 side chains from three RSL molecules each form a salt bridge with a phosphate of the **pctx** cluster (Table 4 aand Figure 5d). In the *P*6_3_ (Figure 5c) and *H*32 cocrystal structures, the packing incorporates “bilayers” of RSL interspersed with **pctx** clusters. Here, Lys25 forms the salt bridges to **pctx**, with the ammonium group approximately equidistant from the three phosphates (Figure 5f). These *exo* interactions between the **pctx** cluster and different lysines of RSL are part and parcel of the distinct crystal packing arrangements obtained. Apparently, the **pctx** cluster acts like a node around which proteins can assemble in different ways depending on which cationic feature (e.g., Lys25 or Lys34) is engaged. Solvent-bound counterions likely contribute further to charge neutralization of **pctx**, but none was evident in the X-ray data.

**Figure 5:**
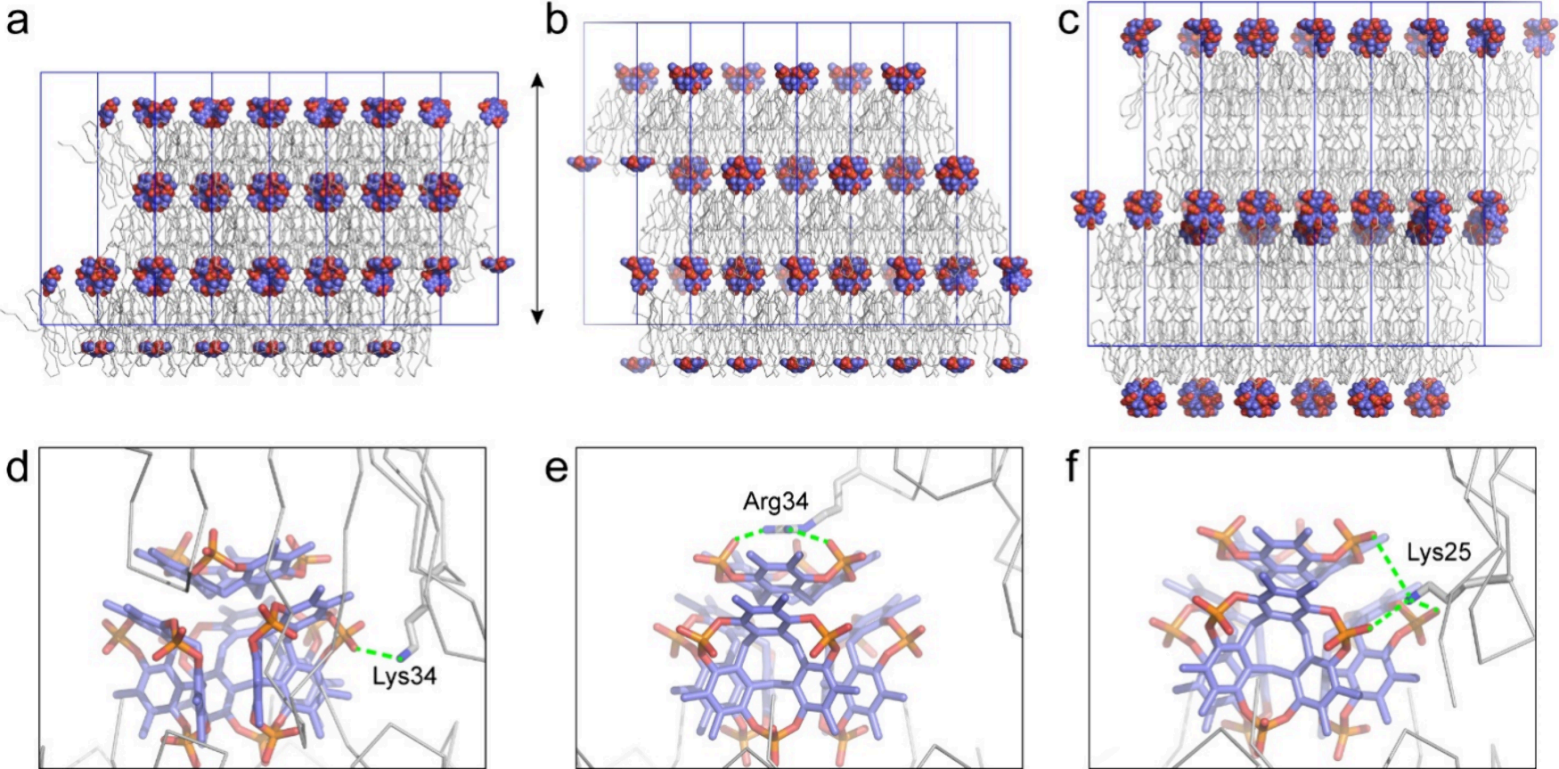
Macrocycle cluster mediated assembly with crystal packing in (a) RSL (space group *H*3), (b) RSL-R_6_ (*H*3) and (c) RSL (*P*6_3_). Shown are **pctx** clusters as spheres, protein as C^α^ traces and unit cell axes in blue. The double-headed arrow indicates a *c* axis of ∼10 nm. (d–f) Protein–cluster interactions corresponding to panels a–c. The detail shows the **pctx** tetrameric cluster (sticks) bound at the N-termini of one RSL trimer (c.f. Figure 4) and the contributions of Lys34, Arg34, or Lys25 from another RSL in the crystal packing. For clarity, only one additional RSL monomer is shown. Green dashed lines indicate salt bridges.

We were intrigued by the distinct binding modes between the phosphate patches of the **pctx** cluster and the lysine side chains in the different cocrystals. With Lys25, there is a neat match between the tetrahedral ammonium group and all three phosphates on a *C*_3_ symmetry axis, while for Lys34 only one phosphate makes a direct salt bridge (Figure 5). These observations prompted us to question how the planar guanidinium^28,29^ of arginine might interact with the cluster. To answer the question we tested the RSL-R_6_ variant, in which all lysines are replaced by arginines.^10^ Remarkably, RSL-R_6_ cocrystallized with **pctx** in the *H*3 space group, similar to RSL. However, instead of the peripheral interaction between Lys34 and the **pctx** cluster (Figure 5d), the guanidinium of Arg34 binds the capping macrocycle (Figure 5e). Apparently, the planar guanidinium has higher affinity for the **pctx** portal than for the *exo* phosphate patch. The consequent rearrangement of the proteins relative to the **pctx** cluster results in a ∼20% elongated *c* axis, and minor contractions of the *a* and *b* axes. Thus, a single residue change, Lys34Arg, leads to expansion of the unit cell in one dimension.

### pctx Is an Arginine Chelator

Besides demonstrating the possibility of crystal engineering, the RSL-R_6_–**pctx** cocrystal structure provides high-resolution detail of arginine complexation by **pctx** (Figures 5 and S3). The guanidinium group is perched 0.8 Å above the plane of the three −P–O^–^ acceptors, with each N atom positioned 2.6 Å from the nearest phosphate oxygen, forming a chelate-type complex^20^ (Table S2). The high charge density of the **pctx** portal likely enables an ion pair contribution to the host–guest complexation of arginine.^30^ Furthermore, each guanidinium N atom is ≤3.4 Å from the nearest phenyl centroid indicative of cation−π bonding.^31^ Overall, the binding mode harks back to the early arginine receptors based on aromatic bis- and trisphosphonates.^20^ In addition to clamping the guanidinium group, the **pctx** receptor engages the alkyl portion of the side chain between two upper rim methyls.

We used this X-ray crystal structure to build a model of **pctx** complexing Lys, with the ammonium positioned equidistant from the phosphate acceptors and from the aromatics (Figure S3). The putative bond lengths are 25–30% longer in the model than in the X-ray structure with arginine (Table S3). This observation suggests that **pctx** is unsuited to binding lysine, consistent with the millimolar host–guest complexation of butylamine, a lysine side chain analogue.^14^

A lysozyme–**pctx** cocrystal structure provides further evidence for arginine selectivity (Figure 6). Lysozyme contains 11 arginines and 6 lysines including the N-terminus Lys1. This protein is readily crystallizable and of the >1000 PDB entries, 88% are in space group *P*4_3_2_1_2. Interestingly, this crystal form can accommodate lysozyme–ligand complexes with relatively large components such as crystallophore (MW_*t*_ 560 Da) or adenosine triphosphate (MW_*t*_ 507 Da).^32,33^ Therefore, we were surprised to find the lysozyme–**pctx** cocrystal structure is trigonal in space group *P*3_1_21. This rare space group for lysozyme was attributed previously to the precipitant action of magnesium sulfate.^34^ In the structure, **pctx** complexes Arg73 (Figure 6b) with the same binding mode as observed in the cocrystal structure with RSL-R_6_ (Figure S3). The binding site also includes the guanidinium of Arg61 forming a salt bridge with one of the phosphates. A second **pctx** demonstrates an alternative binding mode. Here, the side chain of Arg112 is oriented perpendicular to the plane of the phosphate acceptors (Figure 6c). While the macrocycle does not form clusters in this structure, the Arg73 binding site is at a *C*_2_ crystallographic axis such that two molecules of **pctx** make van der Waals contact.

**Figure 6:**
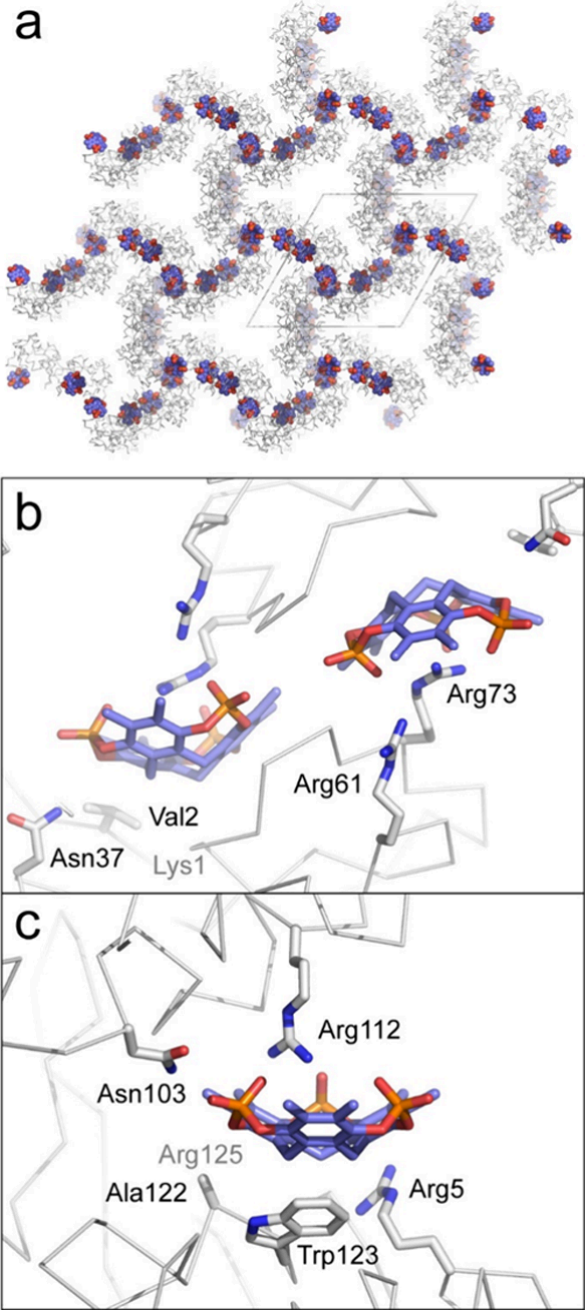
Lysozyme–**pctx** cocrystal structure. (a) Crystal packing in space group *P*3_1_21. (b) **pctx** binding at Arg73, on a *C*_2_ crystallographic axis. (c) Peripheral **pctx**–Arg112 complexation. Other interacting side chains shown as sticks. The approximate locations of Lys1 (panel b) and Arg125 (panel c) are indicated.

Lys1 is near the **pctx** binding site (Figure 6b). However, both of the Lys1 ammonium groups are tucked away forming intramolecular noncovalent bonds within lysozyme.^18^ Apparently, the stability of this tertiary structure feature outweighs any free energy gain possible in Lys1–**pctx** complexation. The ∼90 Å^2^ accessible surface area of Lys1 is significantly lower than the buried surface area of Ser1 (115 Å^2^) in the RSL–**pctx** structure. The inaccessibility of Lysozyme Lys1 is also suggested by the lack of reaction with dansyl chloride.^35^ Solution state NMR spectroscopy provides further evidence of **pctx** binding to arginine rather than the N-terminus of Lysozyme. Using ^1^H–^15^N HSQC measurements (Figure S4) and the known resonance assignments,^36^ we identified Arg125 as the principal binding site for **pctx**. Chemical shift perturbations for the resonances of Val120, Ala122, Trp123 (indole), Leu124, Arg125 and Gly126 suggest **pctx** complexation of Arg125 (Δδ ^1^H ∼ 0.08 ppm, Figure S4). Interestingly some of these residues occur in the crystallographically defined binding site at Arg5 (Figure 6c), which also has a small chemical shift perturbation. Possibly, **pctx** preferentially binds Arg125 in solution but has higher affinity for the Arg5/Trp123 site in the solid state due to the additional interactions arising in the crystal packing, such as the Arg112 contribution. The resonances of Val2 and Phe3 were unperturbed suggesting that the N-terminus is not a binding site (Figure S4).

A **pctx**–Tris cocrystal structure lends further insights into host–guest chelation by the phosphocavitand. Drops containing 1 mM protein and 20–50 mM **pctx** yielded small molecule crystals in several conditions. Crystals from a PEG/ammonium sulfate mixture were solved in the triclinic system *P*1̅. In this structure (CCDC 2442403, Figure S5), despite the presence of >0.3 M ammonium counterions, the *C*_3_-symmetric **pctx** accommodates a Tris guest originating from the protein sample. Some features of the Tris binding are similar to Ser1 encapsulation (Figure 4a). Equivalent hydrogen bonds occur between each of the phosphates and the three hydroxyls of Tris (**pctx**–P–O···O–Tris <2.7 Å). There are also three equivalent CH−π bonds between the Tris methylenes and the aromatic rings (Tris–C···centroid = 3.5 Å). There is a cation-π character to the latter interaction.^31^ Unlike the Ser1-N^α^, the Tris ammonium group points away from the **pctx** cavity and engages in hydrogen bonding with the hydroxyl group of another Tris and two water molecules. The Tris ammoniums and water molecules form a hydrogen bond network to the phosphates of **pctx**. Seemingly, the symmetry matched^37^ chelate-type complex^20^ takes precedence over a possible salt bridge between the ammonium and a **pctx**-phosphate.

### Protein–pctx–Zn^2+^ Assemblies

The RSL–**pctx** cocrystals obtained in the presence of 0.2 M zinc acetate (Figure 3c) were solved in space group *H*32 with a similar structure to the *P*63 crystals (Figure 5) but including 8 zinc ions in the asymmetric unit. Zinc single-wavelength anomalous dispersion (SAD)^38^ experiments confirmed the positions of these ions (Figure S6). Remarkably, the **pctx** cluster bearing 12 phosphates is compatible with zinc complexation (Figure 7a). Ser1, encapsulated by **pctx**, coordinates Zn^2+^ via the N-terminal amino and the backbone carbonyl (Ser1-N^α^···Zn = 2.2 Å and Ser1-CO···Zn = 2.7 Å). The amine of Ser1 is also bonded to the macrocycle (Ser1-N^α^···O–P-**pctx** = 2.6 Å) similar to that described in Figure 4 and Table 3. Two water molecules complete the Zn^2+^ coordination sphere. While this zinc does not bind the macrocycle directly, there is an attractive charge–charge interaction with the **pctx** phosphate via Ser1. In addition to the zinc ions binding to each Ser1, a fourth Zn^2+^ coordinates three phosphates of the macrocycle cluster at a 3-fold symmetry axis (Figure 7a). These data suggest the possibility of complementing protein–**pctx** clusters with metal ions for aiding structure determination or catalysis.^2,32^

**Figure 7:**
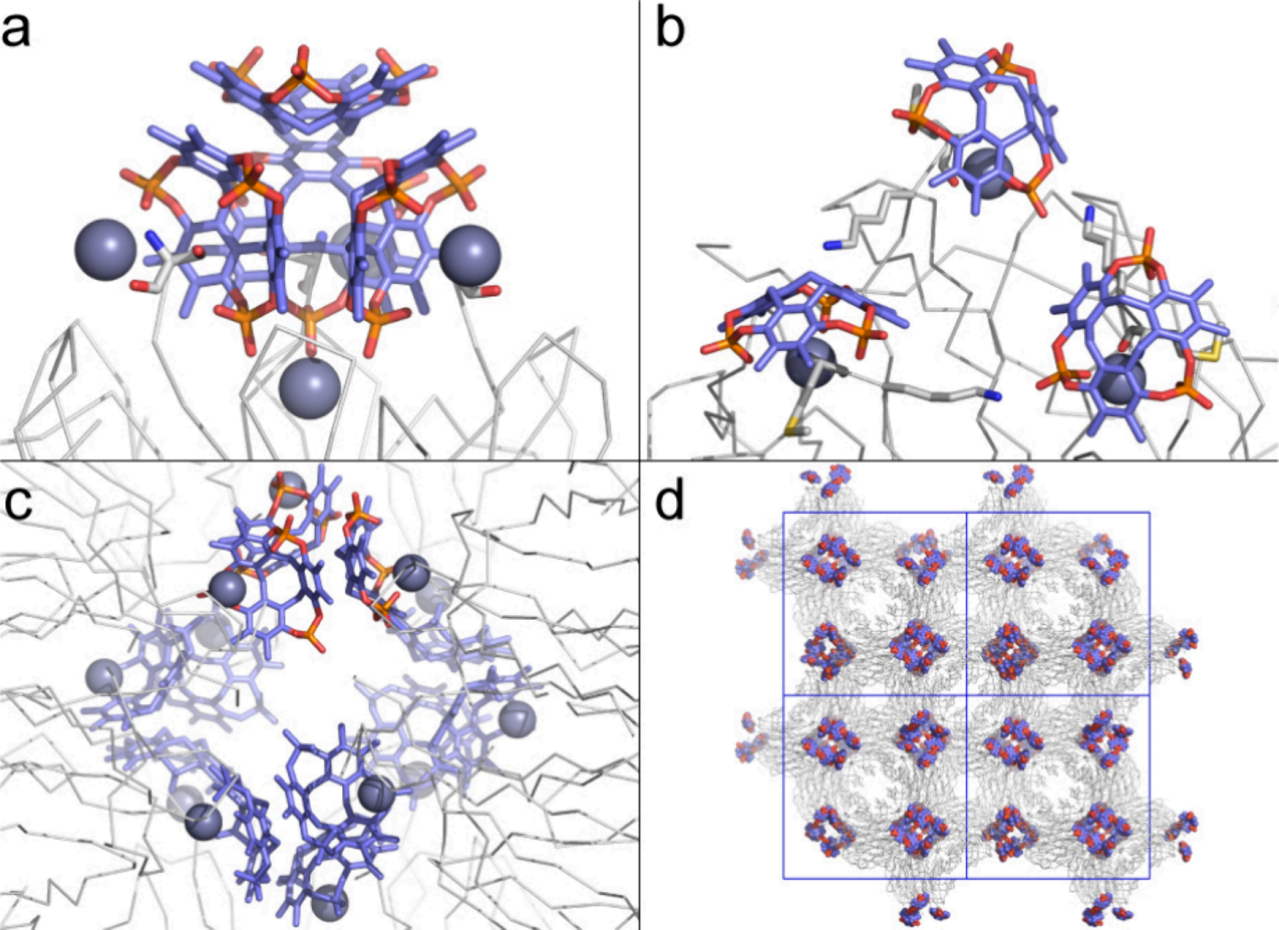
Protein–**pctx**–Zn^2+^ cocrystals. (a) The **pctx** cluster bound to RSL and zinc (space group *H*32). Note the fourth zinc ion binding to three phosphates at the *C*_3_ symmetry axis. Zinc ions and Ser1 shown as spheres and sticks, respectively. (b) In MK-RSL, each N-terminus binds one zinc and one **pctx**. Met0 and Lys1 shown as sticks. (c) Macrocycle clustering arises as four MK-RSL–**pctx**–Zn complexes (shown in b) assemble with tetrahedral geometry, forming a cage of 12 macrocycles. One of the four trimeric **pctx** clusters is in full-color. (d) The overall MK-RSL–**pctx**–Zn crystal packing (space group *P*4_1_2_1_2) viewed along the *c* axis, illustrating the macrocycle and protein cages. **pctx** and zinc represented as spheres, proteins as C^α^ traces and unit cell axes in blue.

Zinc complexation takes on another aspect in the assembly with MK-RSL (Figure 7). This variant of RSL has a cationic, extended and disordered N-terminus containing Met0 and Lys1 that functions as a macrocycle binding site.^5,19,39^ MK-RSL–**pctx**–Zn cocrystals grew in the same conditions as the RSL–**pctx**–Zn cocrystals (Table 2), but in space group *P*4_1_2_1_2 and involving a different type of **pctx** cluster. Each of the three N-terminal methionine residues coordinates a Zn^2+^ ion,^21^ and each Met–Zn complex is encapsulated by a **pctx**. The penta-coordinate Zn^2+^, confirmed by Zn SAD experiments (Figure S6), binds the N-terminal amino and carbonyl groups (Met0-N^α^···Zn = Met0-CO···Zn ≤ 2.1 Å), a phosphate (**pctx**–P–O···Zn = 2.0 Å), a carboxylate (Asp46-O^δ^···Zn = 2.0 Å) and a water molecule. The metal–amino complex binds **pctx** via salt bridges and cation−π bonds. In addition to the N^α^ and CO, the C^α^ and C^β^ of Met0 are encapsulated and form CH−π bonds with the **pctx** cavity. The thioether portion of Met0 remains outside the macrocycle. Lys1 makes numerous *exo* interactions with **pctx** (Figure 7d), including a van der Waals contact (Lys1-C^β^···C-**pctx** = 3.6 Å), a hydrogen bond (Lys-N^α^···O–P-**pctx** = 2.7 Å) and salt bridges. The side chain ammonium extends away to salt bridge with two phosphates in an adjacent **pctx** cluster (Lys-N^ζ^···O–P-**pctx** = 3.1–3.3 Å). This interaction is similar to the roles played by Lys25/Lys34 in the RSL–**pctx** structures (Figure 5).

In contrast to the tetrameric clusters in cocrystals with RSL, the cocrystal with MK-RSL incorporates trimeric clusters of **pctx**. The trimer is an approximately trigonal planar arrangement of **pctx** held together by coplanar π–π stacking (centroid···centroid = 3.6 Å) and CH−π bonds with the methylene bridges. Each component of the cluster binds a different MK-RSL trimer and four such trimeric clusters coalesce forming a supramolecular cage of macrocycles (Figure 7c,d). The resulting tetrahedral assembly of MK-RSL is a porous structure with 50% solvent content, comprising protein cages and protein–Zn–macrocycle cages. The latter can be described as a Zn_12_ **pctx**_12_(protein)_4_ assembly, with similarities to metal organic cages.^8,22^

## Discussion

Similar to **sclx**_**4**_ and prototypical arginine receptors,^17,20^ the physicochemical properties of **pctx** ^14^ (Figure 1 and Table 1) inspired us to investigate protein recognition and assembly by this novel phosphocavitand. A cocrystal structure and a solution NMR study confirmed that **pctx** binds arginine in lysozyme (Figures 6 and S4). The Arg side chain complements the **pctx** receptor with size and charge matching between the guanidinium and the three portal phosphates (Figures 5e, 6, and S3). In contrast, there was no evidence for lysine encapsulation suggesting that, unlike **sclx**_**4**_ and the molecular tweezers,^17,18,25^ **pctx** is selective for arginine. Apparently, this selectivity arises from the complementarity of the guanidinium (but not the ammonium) with the **pctx** portal phosphates. A cocrystal structure with MK-RSL, containing the highly accessible Lys1, emphasizes the lack of lysine encapsulation by **pctx**. Selective Arg binding is desirable considering the importance of arginine and the cation−π bond in protein–protein interfaces.^28,31^ **pctx** could be used as a protein modulator, for example, with peptide appendages that enhance the interaction capability.^26^

The experiments with lysozyme hint at significantly lower binding affinity of **pctx** compared to **sclx**_**4**_. Mixtures of lysozyme and up to 5 mM **pctx** are soluble, while even micromolar concentrations of **sclx**_**4**_ precipitate lysozyme.^18^ Another interesting contrast is that lysozyme–**sclx**_**4**_ complexation involves the two most solvent exposed arginines, Arg128 and Arg14. The **pctx** binding sites include Arg5, Arg61, Arg73, Arg112 and Arg125, all of which are less accessible (Figure S7) than the residues bound by **sclx**_**4**_. Geometric constraints may be responsible for these differences in arginine binding. The shallow **pctx** may select “buried” arginine side chains, as *exo* interactions are possible with the surrounding residues (Figure 6). Highly accessible arginine side chains that protrude into the solvent lack additional contributions to the binding enthalpy as there are no neighboring groups for *exo* complexation.

RSL–**pctx** cocrystal structures revealed the surprising result of N-terminal recognition. Such binding is not possible with an inaccessible N-terminus, as in lysozyme. The small, polar Ser1 of RSL proves to be a suitable guest as **pctx** both encapsulates the side chain and forms a salt bridge with the ammonium group. While Ser1 dominates the complex, Ser2 and Gln4 each contribute a hydrogen bond. Aspects of the Ser1–**pctx** complex are similar to (1) serine encapsulation by a tetra-phosphonate cavitand (CCDC 1415492)^24^ and by **sclx**_**4**_ (CCDC 202923),^40^ and (2) cucurbituril interactions with N-terminal aliphatic or aromatic residues (e.g., CCDC 628234).^5^ Receptors that specifically bind the N-terminus are advantageous as binding is conferred by a few residues rather than a protein surface patch.^5,19,39^ Such site selectivity means that proteins with a 1–3 residue “tag” are amenable to purification, sensing, assembly *etc* by the receptor.^5^ It appears that compared with the larger and deeper **sclx**_**4**_, the dimensions of **pctx** are favorable for Ser1 encapsulation (including enthalpically favorable interactions with surrounding residues). The structure with MK-RSL further suggests a preference for small residues as the thioether portion of the methionine side chain is outside the **pctx** cavity. Future studies will determine what other N-terminal residue types **pctx** can complex/encapsulate. Interestingly, protein–**pctx** complexation is compatible with Zn^2+^ chelation at the N-terminus, enabling dual metal- and macrocycle-mediated assembly (Figure 7). Zinc is highly prevalent in proteins and is the metal of choice for directing protein assembly, including protein-based MOFs.^8,9,21,38^

The salient result with the RSL–**pctx** cocrystals is the occurrence of macrocycle clusters. A ∼2.5 kDa tetrahedral cluster of the phosphocavitand, approximating a dimpled sphere with shallow cavities and a formal net charge of minus 12, sits in the channel at the narrow end of RSL (Figure 4). The three macrocycles at the base of the cluster bind to the three cationic N-termini, and the fourth capping macrocycle is empty. **pctx** recognition of the N-terminus, and concomitant clustering effectively plugs the RSL toroid. Previously, a designed 6-bladed β-propeller (Pizza, PDB 5CHB) was plugged with a cadmium chloride nanocrystal.^27^ And we have shown that sulfonato-calix[8]­arene can block the wide end of the designed β-propeller Pent (PDB 8R3B).^41^ Together these structures suggest strategies for modulating the β-propeller fold which is widespread in different enzyme classes.

In cocrystals with RSL, three additional proteins interact *exo* to the **pctx** cluster. Different assemblies form depending on which lysine residues engage the cluster (Figure 5). Replacing all three lysines of RSL with arginine, results in selective complexation of Arg34 by the capping macrocycle and consequent reorganization of the crystal packing. Thus, crystal engineering was possible by a single residue modification that altered protein binding to the macrocycle cluster. As polymorph selection occurs at the earliest stages of protein crystallogenesis,^42^ it is likely that protein–**pctx** clusters (Figure 4b) act as precursors for crystal nucleation. Furthermore, it appears that the multivalency of RSL and collocation of the three N-termini are propitious to cluster formation. In MK-RSL, the longer N-termini result in a different type of clustering that depends to a lesser extent on multivalency (compare Figures 4b and 7b). The cocrystals of MK-RSL and **pctx** also include zinc, which mediates macrocycle binding (Met–Zn^2+^–**pctx** complex). In the crystal, four complexes of MK-RSL laden with zinc and **pctx** coalesce in a tetrahedral geometry with trimeric **pctx** clusters at the vertices (Figure 7c). The cage-like substructure of macrocycles, similar to metal organic cages,^22^ has an internal diameter of ∼2 nm. The voids in the protein assembly also have an internal diameter of ∼2 nm (Figure 7d). This porous material relies on the N-terminal Met-Lys motif combined with zinc chelation, **pctx** encapsulation and macrocycle clustering. Including the Met-Lys motif in other proteins may provide a straightforward route to new types of materials directed by Zn^2+^–**pctx** complexation.

While **pctx**–lysozyme complexation and arginine binding are evident by NMR spectroscopy (Figure S4), similar experiments with RSL or MK-RSL did not yield binding in solution (data not shown). Under the conditions tested, **pctx** interaction with the neutral RSL may be too weak for detection, while **pctx** binding to the cationic and arginine-rich lysozyme is sufficient. These contrasting data suggest that in solution **pctx**–arginine complexation (chelation) is more favorable than **pctx** encapsulation of the N-terminal serine (one salt bridge). There is also a disparity in protein binding between **sclx**_**4**_ and **pctx**, which may be attributed to differences in the macrocycle cavity volume. For example, with MK-RSL, **sclx**_**4**_ encapsulation of the N-terminal Met is evidenced in both a crystal structure (PDB 9GR3) and an NMR study.^19^ In this case **sclx**_**4**_ wholly encapsulates the thioether side chain. As the expulsion of water from the cavity (hydrophobic effect) is the driving force for host–guest encapsulation,^30,43^ weaker binding is expected with **pctx**, which has a shallow cavity (depth ∼ 3 Å) compared to **sclx**_**4**_ (∼6 Å, Table 1). This difference may partly explain why micromolar **sclx**_**4**_, in contrast to millimolar concentrations of **pctx**, induce lysozyme precipitation.

The hydrophobic base of **pctx** (Figure 1) is conducive to cluster formations that are not possible with **sclx**_**4**_, with major implications for protein assembly. Evidence is accumulating in favor of protein crystal engineering by discrete macrocycle oligomers or supramolecular synthons.^44,45^ The tetrameric **pctx** cluster appears to be a pivotal example, acting as an assembly node with four shallow cavities for single residue encapsulation and four *exo* anionic sites for complexing additional cationic residues or metal ions. While discrete capsules or cages of calixarenes rely on intermolecular hydrogen bonding or metal chelation,^46,47^ aromatic macrocycle assembly more often results in π-stacked supramolecular polymers (open/infinite assemblies). Examples of π-stacked discrete oligomers (closed assemblies) are comparatively rare and in some cases are controlled by guest binding, including protein complexation.^46–51^ In **pctx**, the tetrahedral closed assembly arises from back-to-back π-stacking, as originally observed with the related macrocycle cyclotriveratrylene.^52,53^ The coplanar π–π stacking of **pctx** (centroid···centroid = 3.7 Å) buries ∼245 Å^2^ per macrocycle suggesting a significant contribution of the hydrophobic effect to cluster stability. While **pctx** has intrinsically lower binding affinity compared to **sclx**_**4**_, the self-assembly capabilities of **pctx** confer more interesting possibilities for fabricating protein-based materials.

## Conclusions

The noncovalent modification of proteins and the fabrication of (porous) assemblies have diverse applications from biopharmaceuticals to biomaterials.^1–5^ This first study of **pctx**–protein interactions demonstrates selectivity for arginine or for small and accessible N-terminal residues. The phosphocavitand, readily synthesized in three steps,^14^ is a potentially valuable modulator of protein–protein interfaces which frequently involve arginine residues. The modification of **pctx** with additional protein binding features (such as peptide appendages) may be necessary, and will benefit from existing synthetic strategies.^26,54^ The ease of introducing a Met-Lys N-terminal motif into proteins of interest, suggests a route to Zn^2+^/**pctx** controlled assembly. More generally, the N-terminal complexation and the alternative **pctx** cluster formations (trimeric versus tetrameric) indicate a promising versatility and scope for assembling different protein types. In some crystal structures with tetrameric clusters, only three of the cavities are utilized, while the capping macrocycle is empty. Arginine complexation by the capping macrocycle (in cocrystals with RSL-R_6_) suggests the possibility of heterogeneous assemblies where different protein types with distinct binding features assemble on the same cluster. It is easy to envisage arrangements with only two or all four cavities utilized for protein binding, as well as the possibility of one or more cavities accommodating other guest types.^55^ Future research will focus on (1) the repertoire of N-terminal residues that are suitable guests, (2) how protein–**pctx** interactions lead to macrocycle clusters that mediate assembly/nucleate crystallization and (3) mixed protein assemblies on **pctx** clusters.

## Supplementary Material



## Notes

Methods; X-ray data statistics; noncovalent bond lengths; structure diagrams; HSQC spectra; and Zn SAD Fourier maps (PDF)
