# Supplementary material for: Protein Recognition and Assembly by a Phosphocavitand
Source: J Am Chem Soc. 2025 Jul 22;147(31):28107–16. doi: 10.1021/jacs.5c08121 (PMC12333348; doi:10.1021/jacs.5c08121)
Supplement: Supplementary file 1 [file ja5c08121_si_001.pdf]

SUPPORTING INFORMATION

Protein Recognition and Assembly by a Phosphocavitand

Colin P. Wren,[a] Ronan J. Flood,[a] Niamh M. Mockler,[a] Martin Savko,[b] Maura Malinska,[c] Qiang Shi,[d] and Peter B. Crowley*[,a]

[a]School of Biological and Chemical Sciences, University of Galway, Galway, H91 TK33, Ireland.

[b]Synchrotron SOLEIL, L'Orme des Merisiers, Saint-Aubin BP 48, 91192 Gif-sur-Yvette Cedex, France.

[c]Faculty of Chemistry, University of Warsaw, Pasteura 1, Warsaw, Poland.

[d]Advanced Materials Institute, Qilu University of Technology, Shandong Academy of Sciences, Jinan 250014, China.

*Correspondence to: peter.crowley@universityofgalway.ie +353 91 49 24 80

Keywords: biomaterials; molecular recognition, N-terminus; supramolecular chemistry; Zinc

Contents

Methods		S2
Figure S1	NMR spectra of **pctx**	S4
Table S1	X-ray data collection, processing and refinement statistics	S5
Table S2	X-ray data collection, processing and refinement statistics (lysozyme)	S6
Figure S2	Noncovalent bonds in the Ser1 – **pctx** complex	S7
Figure S3	Arginine encapsulation by **pctx**	S8
Table S3	Noncovalent bond lengths in the Arginine – **pctx** complex	S8
Figure S4	^{1}H-^{15}N HSQC spectra of lysozyme in the absence / presence of **pctx**	S9
Table S3	**pctx** – Tris X-ray data collection, processing and refinement statistics	S10
Figure S5	**pctx** – Tris crystal structure	S11
Figure S6	Zn SAD Fourier maps	S12
Figure S7	Accessible surface areas of arginine/lysine residues in lysozyme	S13
References		S14

Methods

Materials. The ammonium salt of **pctx** was synthesized and characterized by NMR spectroscopy (Figure S1) as described.[1] 100 mM stock solutions of **pctx** were prepared in water and adjusted to pH 7. Commercial D-fructose (Sigma F0127) and lysozyme (Sigma-Aldrich 62971) were used as sourced. RSL, RSL-R_6 and MK-RSL were produced and purified using established methods.[2,3]

Co-crystallization Trials. Sparse matrix screens (JCSG++ HTS, Jena Bioscience) were dispensed with an Oryx8 robot (Douglas Instruments) and incubated at 20 °C. 1 mM RSL or MK-RSL were trialed with 0-50 mM **pctx**. Due to the increased crystallizability of RSL-R_6, these cocrystallization trials were performed at 0.5 mM protein. Samples of RSL and variants included 5 mM D-fructose. 1 mM lysozyme was trialed with 0-5 mM **pctx**. Crystals were optimized by hanging drop vapour diffusion and imaged using an Olympus SZX16 stereomicroscope with an Olympus DP25 digital camera.

X-ray Data Collection, Processing and Model Building. Crystals were cryo-protected in the crystallization solution containing 25-30% glycerol and cryo-cooled in liquid nitrogen. Diffraction data were collected at 100 K at beamline PROXIMA-2A, SOLEIL Synchrotron (Saint Aubin, France) with an EIGER X 9M detector.[4] The data were processed using the autoPROC pipeline,[5] using XDS for integration[6] with scaling and merging in AIMLESS[7] and POINTLESS.[8] Zinc single-wavelength anomalous dispersion (Zn SAD) was collected at a wavelength of 1.28 Å. phenix.Xtriage[9] was used to assess for pathologies, revealing translational noncrystallographic symmetry (tNCS) in the $P6_3$ datasets, with a translation vector of (x, y, 0.5). The structures were solved by molecular replacement in PHASER,[10] using the RSL monomer (PDB 2bt9) as a search model. For MK-RSL, the trimer of MK-RSL (PDB 8c9y) was the search model. For lysozyme, a monomer derived from PDB 4prq was the search model. The **pctx** coordinates and geometric constraints were generated in Grade2.[11] COOT was used to add the coordinates of **pctx** and D-fructose (PDB ID BDF) to the models.[12] Iterative rounds of model building in COOT and refinement in phenix.refine were performed until no further improvements to R_{free} and the electron density could be made. Structures were validated in MolProbity.[13] Protein – ligand interface areas were analysed in PDBePISA.[14]

The structure of the **pctx –** Tris crystal was solved in SHELXT[15] and refined in SHELXL[16] within the Olex2 software suite.[17] Refinement was based on F^2 for all reflections, except those with negative intensities. Weighted *R*-factors (wR) and all goodness-of-fit values (*S*) were based on F^2, whereas conventional *R*-factors were based on amplitudes, with *F* set to zero for negative F^2. The

atomic scattering factors were obtained from the International Tables for Crystallography.[18] Data collection and processing statistics are summarized in Table S4.

Diffuse electron density features (identified as low electron density peaks) were observed in the difference Fourier maps. These features are attributed to water molecules and ammonium cations. Despite multiple trial refinements, attempts to model these features as low-occupancy sites were unsuccessful. Consequently, the data were processed using the PLATON SQUEEZE method[19] prior to final refinement. A solvent mask was calculated, revealing 227 electrons in two voids per unit cell with a total volume of 472 Å^3. This mask is consistent with the presence of 2 × NH_4^+ and 9.5 × H_2O per asymmetric unit, together accounting for 234 electrons per unit cell. The structure is available at CCDC 2442403.

Protein NMR Spectroscopy. Lysozyme – **pctx** interactions were studied by natural abundance 2D ^{1}H–^{15}N HSQC watergate spectra, acquired at 30 °C with 256 scans and 64 increments on a Varian 600 MHz spectrometer with a HCN cold probe. The samples were 2 mM lysozyme with 0 or 4 mM **pctx** in 50 mM sodium acetate, 10% D_2O, pH 4.6. **pctx** interactions with RSL or MK-RSL were tested using 0.1 mM ^{15}N-labelled protein samples in 20 mM potassium phosphate, 50 mM NaCl, 5 mM D-fructose, 10 % D_2O, at pH 4, 5 or 6, and with µL aliquot additions of 100 mM **pctx**.[2,3]

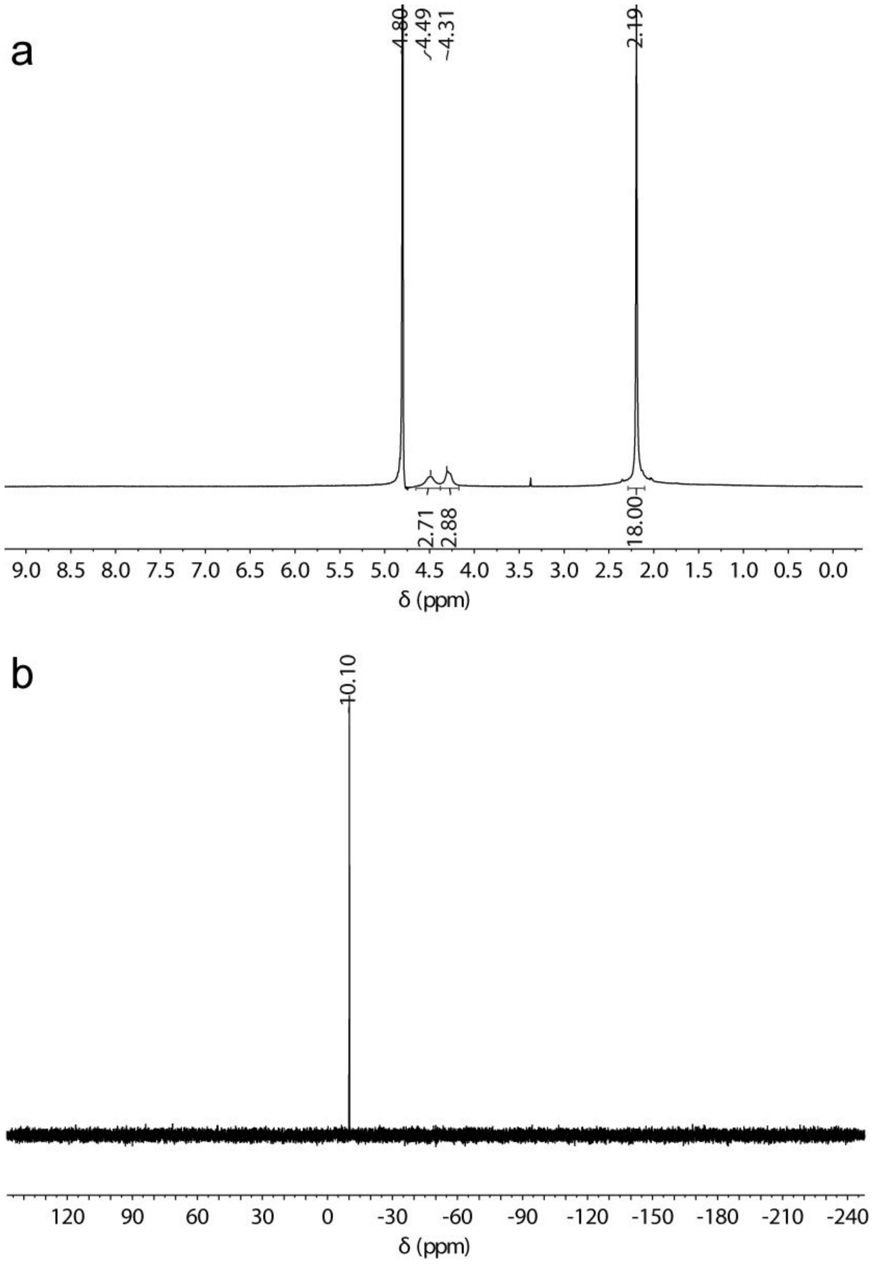

Figure S1. (a) ^{1}H and **(b)** ^{31}P NMR spectra of the **pctx** ammonium salt in D_2O collected at 298 K on a Bruker Avance III 400 MHz NMR spectrometer.

Table S1. X-ray data collection, processing and refinement statistics for RSL, RSL-R_6 and MK-RSL – **pctx** cocrystals.

**Structure**	RSL – **pctx**	RSL-R_6 – **pctx**	RSL – **pctx**	RSL – **pctx**	MK-RSL – **pctx**
**Data Collection**					
**Light Source**	SOLEIL, PROXIMA-2A				
**Wavelength (Å)**	0.98011				
**Space Group**	*H*3	*H*3	*P*6_3	*H*32	*P*4_12_12
**a, b, c (Å)**	46.709	43.569	46.078	45.893	112.158
	46.709	43.569	46.078	45.893	112.158
	102.923	122.997	139.565	421.816	99.312
**α, β, γ (°)**	90.0	90.0	90.0	90.0	90.0
	90.0	90.0	90.0	90.0	90.0
	120.0	120.0	120.0	120.0	90.0
**Resolution (Å)**	37.65 – 1.04	18.65 – 1.49	69.78 – 1.07	39.06 – 1.49	61.97 – 1.50
	(1.06 – 1.04)	(1.52 – 1.49)	(1.09 – 1.07)	(1.52 – 1.49)	(1.52 – 1.50)
**# reflections**	279370	144586	1144600	494332	2728386
	(173)	(4873)	(6937)	(15730)	(105492)
**# unique reflections**	32889	14063	71473	28854	102148 (5025)
	(166)	(666)	(2731)	(1339)	
**Multiplicity**	8.5 (1.0)	10.3 (7.3)	16.0 (2.5)	17.1 (11.7)	26.7 (21.0)
**I/σ (I)**	12.7 (2.1)	25.6 (5.2)	10.6 (0.6)	24.9 (6.5)	17.4 (2.0)
**Completeness (%)**	81.2 (88.3)	99.6 (94.2)	97.8 (75.9)	99.8 (95.4)	100.0 (100.0)
**R_{meas} (%)**	10.0 (20.4)	5.4 (29.7)	11.1 (147.1)	7.5 (32.1)	14.2 (162.1)
**R_{pim} (%)**	3.3 (14.4)	1.7 (10.6)	2.5 (86.3)	1.8 (8.9)	2.7 (34.7)
**$CC_{1/2}$**	0.993 (0.943)	0.999 (0.983)	0.999 (0.334)	0.999 (0.955)	0.999 (0.613)
**Solvent Content (%)**	40	42	39	39	50
**Phasing Statistics**					
**TF Z-score**	10.3	8.4	14.9	11.5	11.4
**Log-likelihood Gain**	145	204	319	226	221
**Refinement**					
**R_{work}**	16.84	17.15	16.87	18.10	17.58
**R_{free}**	17.39	20.28	18.37	20.63	19.88
**rmsd bonds (Å)**	0.004	0.006	0.009	0.004	0.016
**rmsd angles (°)**	1.171	1.301	1.338	1.149	1.589
**# molecules in asymmetric unit**					
**Protein chains**	1	1	2	2	6
**pctx**	2	2	4	4	6
**water**	171	109	258	201	630
**Zinc**	-	-	-	8	6
**Ave. B-factor (Å^2)**	9.73	15.99	13.92	17.10	17.09
**Clashscore**	0	1.34	0.34	2.04	1.85
**Ramachandran analysis, % residues in**					
**favoured regions**	100.0	96.59	97.16	97.73	97.75
**allowed regions**	0.0	3.41	2.84	2.27	2.25
**PDB id**	9HRV	9HRZ	9HRW	9HRX	9HRY

Table S2. X-ray data collection, processing and refinement statistics for a lysozyme – **pctx** cocrystal.

**Data Collection**	
**Light Source**	SOLEIL, PROXIMA-2A
**Wavelength (Å)**	0.98011
**Space Group**	$P3_121$
**a, b, c (Å)**	86.307<br>86.307<br>72.738
**α, β, γ (°)**	90.0<br>90.0<br>120.0
**Resolution (Å)**	52.13 – 1.88<br>(1.91 – 1.88)
**# reflections**	449045 (23027)
**# unique reflections**	25883(1268)
**Multiplicity**	17.3 (18.2)
**I/σ (I)**	16.6 (2.3)
**Completeness (%)**	100 (100)
**R_{meas} (%)**	11.1 (159.5)
**R_{pim} (%)**	2.7(37.4)
**$CC_{1/2}$**	0.998 (0.849)
**Solvent Content (%)**	53
**Phaser Analysis**	
**TF Z-score**	12.7
**Log-likelihood Gain**	119
**Refinement**	
**R_{work}**	19.13
**R_{free}**	22.41
**rmsd bonds (Å)**	0.008
**rmsd angles (°)**	1.113
**# molecules in asymmetric unit**	
**Protein chains**	2
**pctx**	2
**water**	106
**Ave. B-factor ($Å^2$)**	36.60
**Clashscore**	1.97
**Ramachandran analysis, % residues in**	
**favoured regions**	99.61
**allowed regions**	0.39
**PDB id**	9HRU

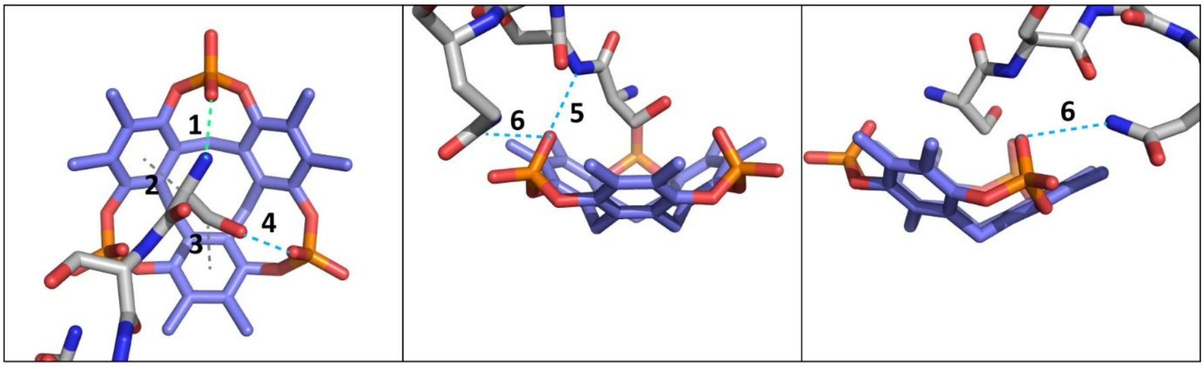

#	Residue-atom	pctx	Distance (Å)	Interaction
**1**	Ser1-N^{α}	O-P^1	2.7	Salt bridge
**2**	Ser1-C^{α}	centroid	3.8	CH-π (cation-π)
**3**	Ser1-C^{β}	centroid	3.4	CH-π
**4**	Ser1-O^{γ}	O-P^2	2.5	Hydrogen bond
**5**	Ser2-N^{α}	O-P^3	2.8	Hydrogen bond
**6**	Gln4-N^{ε}	O-P^3	2.9	Hydrogen bond

Figure S2. Different views of the Ser1 – **pctx** complex with noncovalent bonds indicated as dashed lines. The table enumerates each bond type.

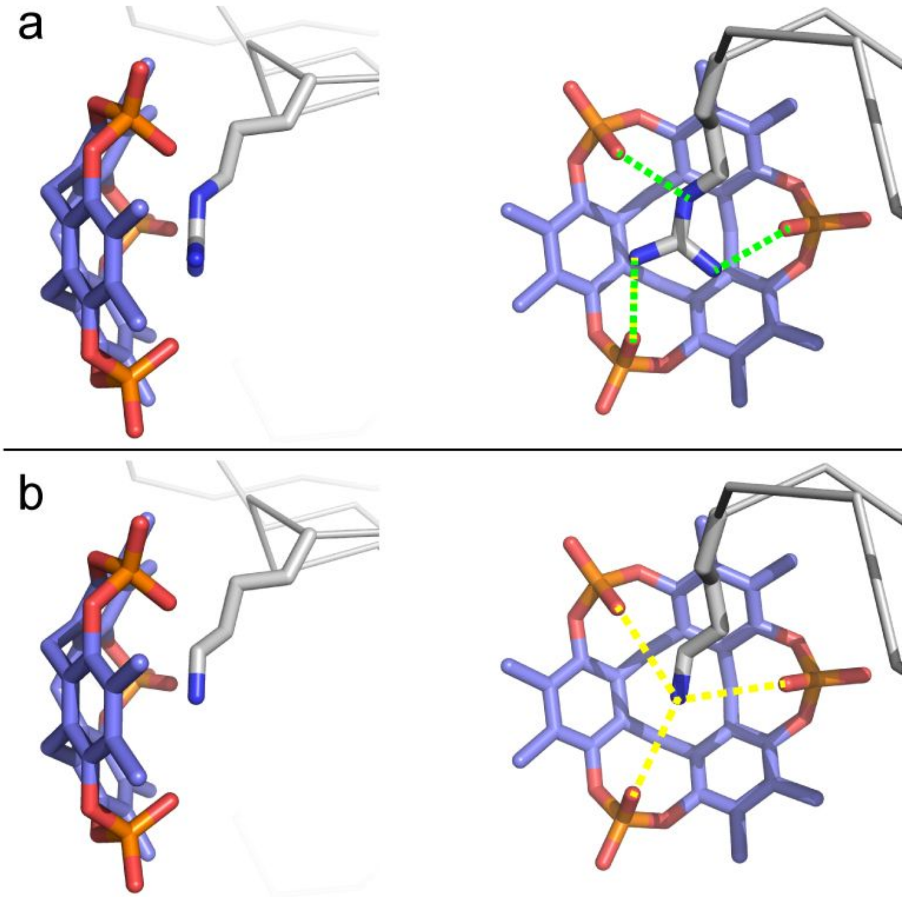

Figure S3. (a) Arginine (Arg34) encapsulation by **pctx** in the 1.5 Å resolution cocrystal structure with RSL-R_6. Green dashed lines indicate salt bridges. **(b)** A model of lysine encapsulation with yellow dashed lines indicating ammonium – phosphate bonds. Refer to Table S3 for bond details.

Table S3. Arg – **pctx** noncovalent bond lengths in the cocrystal structure with RSL-R_6, and putative bond lengths derived from the lysine model (Figure S3).

Residue-atom	pctx	Distance (Å)	Interaction
Arg34-N^{ε}	O-P^{1}	2.6	Salt bridge
Arg34-N^{ε}	centroid^{1}	3.4	cation-π
Arg34-$N^{\eta 2}$	O-P^{2}	2.6	Salt bridge
Arg34-$N^{\eta 2}$	centroid^{2}	3.3	cation-π
Arg34-$N^{\eta 1}$	O-P^{3}	2.6	Salt bridge
Arg34-$N^{\eta 1}$	centroid^{3}	3.3	cation-π
*Lys34-N^{ζ}	O-P^{1}	3.4	Salt bridge
*Lys34-C^{ε}	centroid^{1}	3.4	CH-π (cation-π)
*Lys34-N^{ζ}	O-P^{2}	3.4	Salt bridge
*Lys34-N^{ζ}	centroid^{2}	4.1	cation-π
*Lys34-N^{ζ}	O-P^{3}	3.4	Salt bridge
*Lys34-N^{ζ}	centroid^{3}	4.1	cation-π

*Model built by replacing Arg34 with a Lys side chain and retaining high symmetry position.

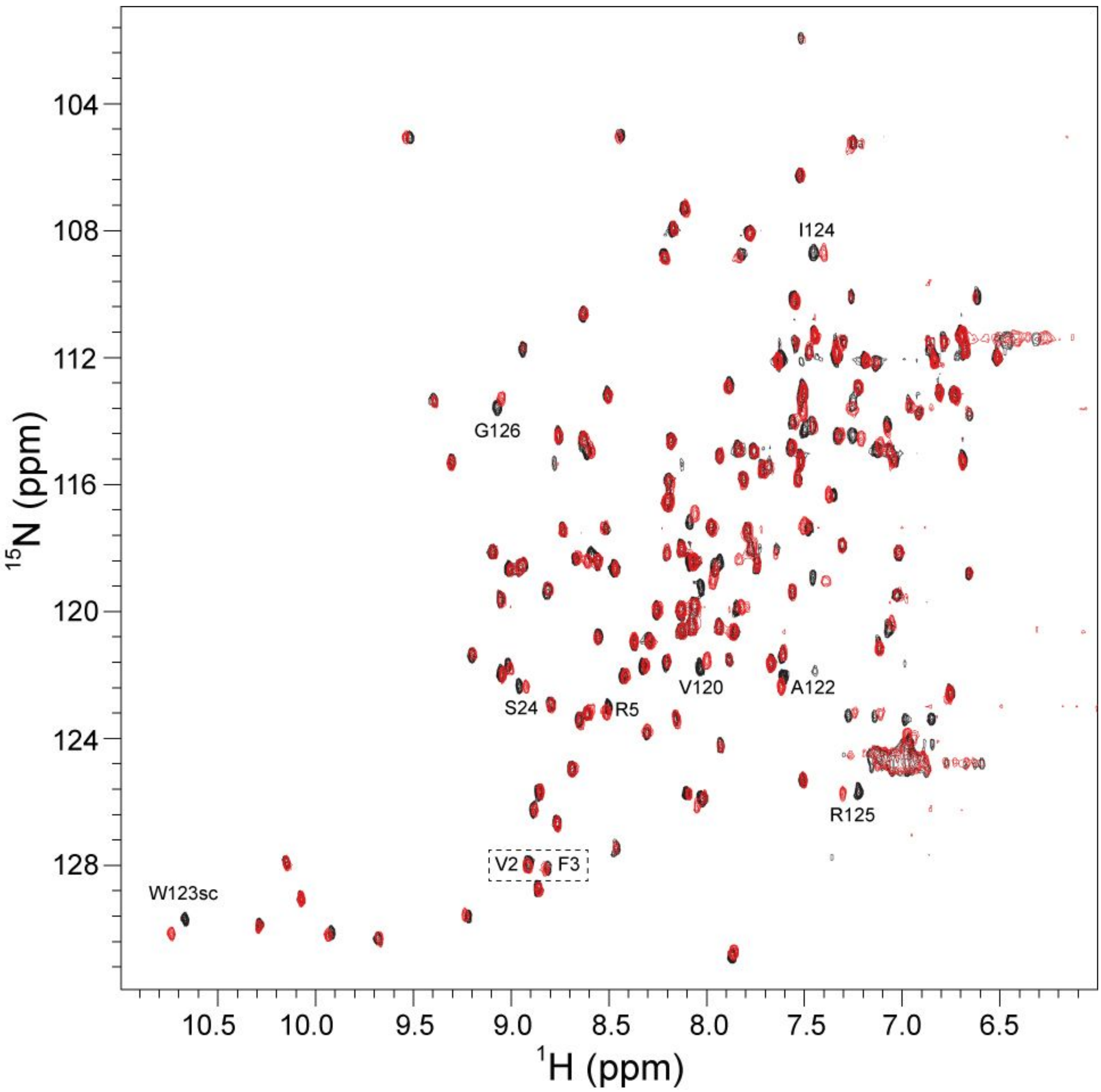

Figure S4. Superposed ^{1}H-^{15}N HSQC spectra of 2 mM lysozyme (black contours) and 2 mM lysozyme plus 4 mM **pctx** (red contours) in 50 mM sodium acetate, 10% D_2O, pH 4.6 at 30 °C. Labels indicate unambiguous resonance assignments. The dashed box highlights the unaffected resonances of Val2 and Phe3, reporters on the N-terminus.

Table S4. X-ray data collection, processing and refinement statistics for a **pctx** – Tris cocrystal.

	**Crystal Data**
**Chemical formula**	$C_{27}H_{24}O_{12}P_3 \cdot C_4H_{12}NO_3 \cdot 6(H_2O) \cdot 2[NH_4^+] \cdot 9.5[H_2O]$
M_r	1070.85
**Crystal system, space group**	Triclinic, $P\bar{1}$
**Temperature (K)**	100
***a*, *b*, *c* (Å)**	13.421 (3), 13.599 (3), 13.936 (3)
**α, β, γ (°)**	106.420 (3), 107.550 (3), 95.850 (3)
V **(Å^3)**	2277.0 (9)
Z	2
**m (mm^{-1})**	0.25
**Crystal size (mm)**	0.2 × 0.2 × 0.1
	**Data collection**
**Radiation type**	SOLEIL synchrotron, PROXIMA-2A, λ = 0.72932 Å
**No. of measured, independent and observed [$I > 2\sigma(I)$] reflections**	9683, 3089, 3066
R_{int}	0.028
θ_{max} **(°)**	21.6
$(\sin \theta/\lambda)_{max}$ **(Å^{-1})**	0.504
	**Refinement**
$R[F^2 > 2\sigma(F^2)]$, $wR(F^2)$, S	0.137, 0.342, 1.19
**No. of reflections**	3089
**No. of parameters**	516
**No. of restraints**	103
**H-atom treatment**	H-atom parameters constrained
	$w = 1/[\sigma^2(F_o^2) + (0.0523P)^2 + 41.2263P]$ where $P = (F_o^2 + 2F_c^2)/3$
ρ_{max}, ρ_{min} **(e Å^{-3})**	0.61, -0.46

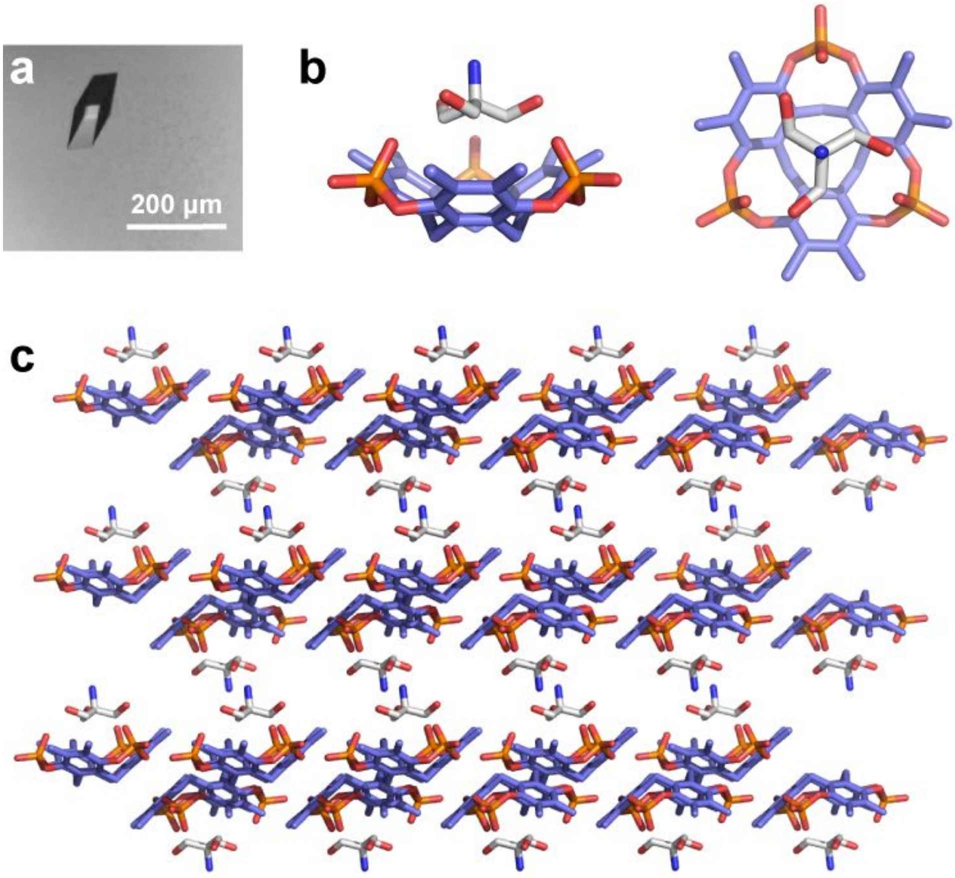

Figure S5. (a) Small molecule crystal obtained in 25.5 % PEG 4000, 15 % glycerol, 0.17 M ammonium sulfate. **(b)** The **pctx** – Tris cocrystal structure, space group $P\overline{1}$, showing two views of the host – guest chelate-type complex. **(c)** Crystal packing viewed along the *c* axis. Water molecules omitted for clarity. Note, Tris was not a constituent of the crystallization condition but originated in the protein sample.

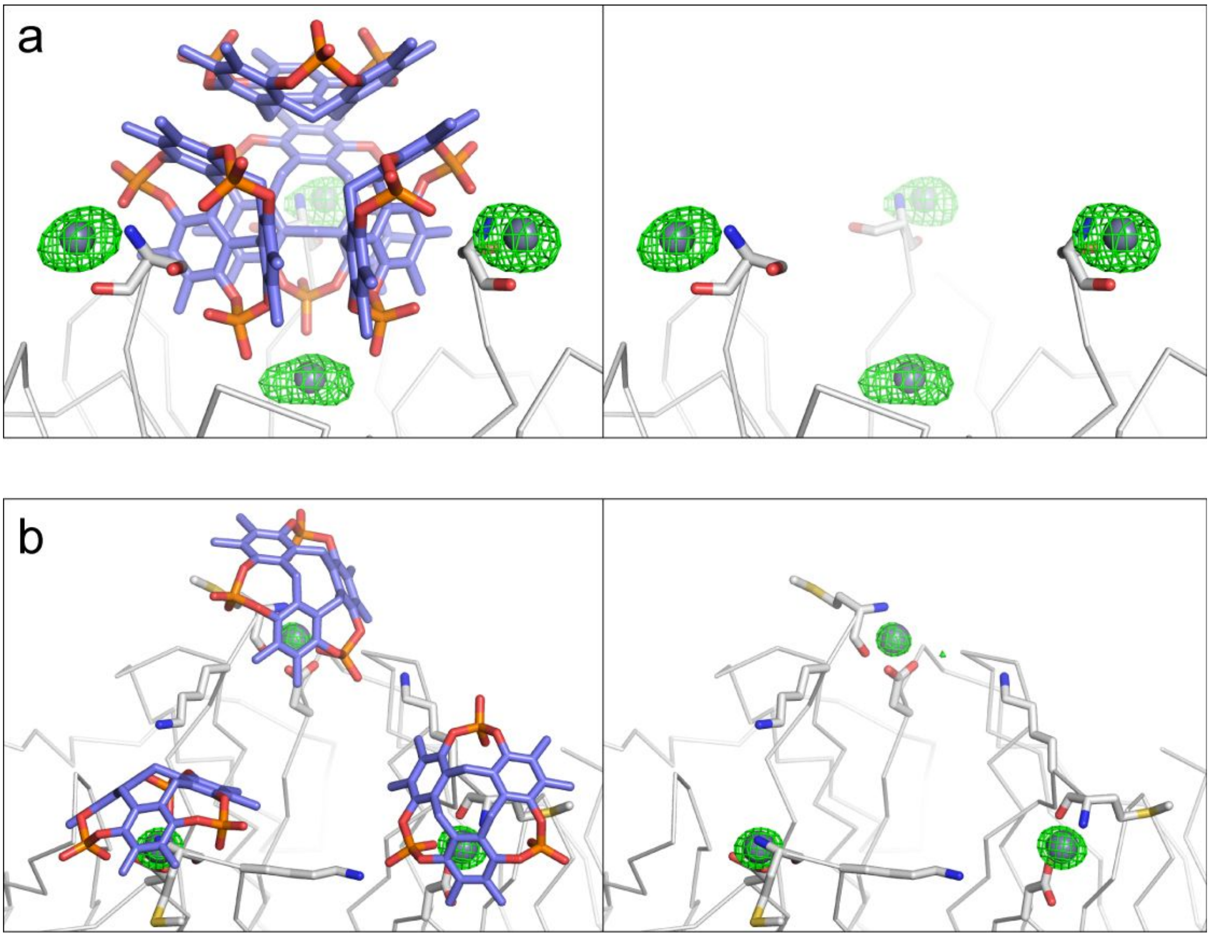

Figure S6. Zn SAD Fourier maps (green mesh contoured at 4.0 σ) reveal the presence of zinc sites in **(a)** RSL and **(b)** MK-RSL – **pctx** cocrystal structures. Protein represented as C^{α} trace, zinc as grey spheres, **pctx** as sticks. N-terminal residues Ser1 (RSL) and Met0 (MK-RSL) shown as sticks. In MK-RSL the side chains of Lys1 and Asp46 are also shown. For clarity, images on right lack **pctx**.

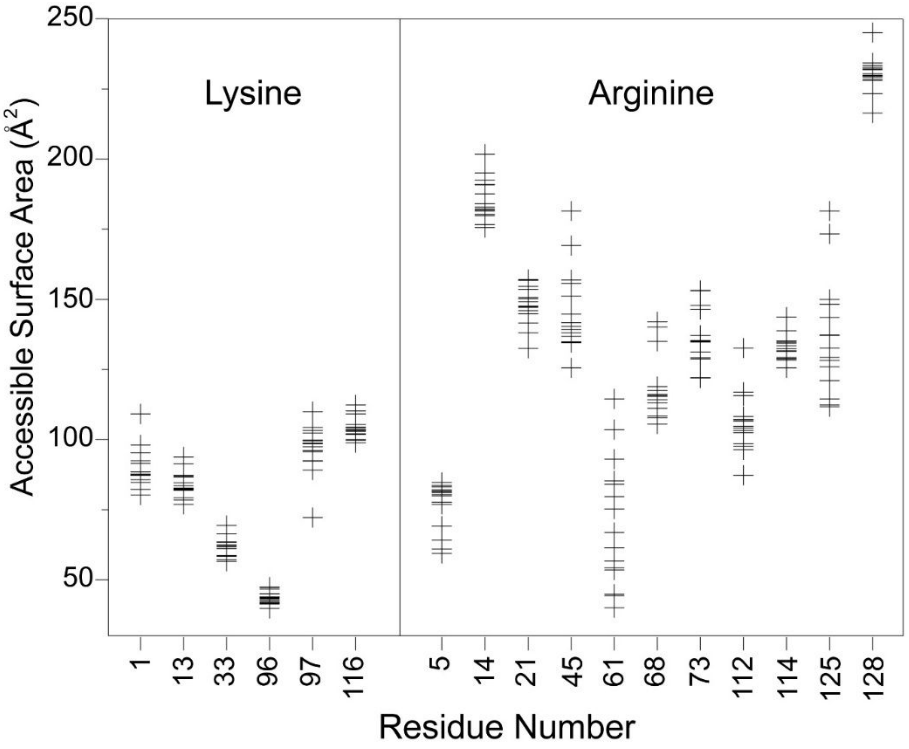

Figure S7. The accessible surface areas of the lysine and arginine residues calculated in 15 high-resolution crystal structures of lysozyme.[20,21]

References

1. Jiao, J.; Sun, G.; Zhang, J.; Lin, C.; Jiang, J.; Wang, L. *Chem. Eur. J.* **2021**, *27*, 16601–16605.
2. Ramberg, K. O.; Engilberge, S.; Skorek, T.; Crowley, P. B. *J. Am. Chem. Soc.* **2021**, *143*, 1896–1907.
3. Ramberg, K. O.; Engilberge, S.; Guagnini, F.; Crowley, P. B. *Org. Biomol. Chem.* **2021**, *19*, 837–844.
4. Polsinelli, I.; *et al. J. Synchrotron Radiat.* **2017**, *24*, 42–52.
5. Vonrhein. C.; *et al. Acta Crystallogr.* **2011**, *D67*, 293–302.
6. Kabsch, W. *Acta Crystallogr.* **2010**, *D66*, 125–132.
7. Evans, P. R.; Murshudov GN. *Acta Crystallogr.* **2013**, *D69*, 1204–1214.
8. Evans PR. *Acta Crystallogr.* **2011**, *D67*, 282–292.
9. Adams, P. D.; *et al. Acta Crystallogr.* **2010**, *D66*, 213–221 (2010).
10. McCoy, A. J.; *et al. J. Appl. Crystallogr.* **2007**, *40*, 658–674.
11. Smart, O. S.; *et al. Grade2 Version 1.5.0*, Global Phasing Ltd (2011).
12. Emsley, P.; Cowtan K. *Acta Crystallogr.* **2004**, *D60*, 2126–2132.
13. Williams, C. J.; *et al. Protein Sci.* **2018**, *27*, 293–315.
14. Krissinel, E.; Henrick, K. *J. Mol. Biol.* **2007**, *372*, 774–797.
15. Sheldrick, G. M. *Acta Crystallogr.* **2015**, *A71*, 3–8.
16. Sheldrick, G. M. *Acta Crystallogr.* **2015**, *C71*, 3–8.
17. Dolomanov, O. V.; Bourhis, L. J.; Gildea, R. J.; Howard, J. A. K.; Puschmann, H. *J. Appl. Crystallogr.* **2009**, *42*, 339–341.
18. Prince, E.; Spiegelman, C. H. Statistical Significance Tests. In *International Tables for Crystallography Volume C: Mathematical, physical and chemical tables*; Prince, E., Ed.; International Tables for Crystallography; Springer Netherlands: Dordrecht, 2004; pp 702–706.
19. Spek, A. L. *Acta Crystallogr. Sect.* **2015**, C*71*, 9–18.
20. McGovern, R. E.; McCarthy, A. A.; Crowley, P. B. *Chem. Commun.* **2014**, *50*, 10412–10415.
21. McGovern, R. E.; *et al. Chem. Sci.* **2015**, *6*, 442–449.
